# ALVAC-HIV and AIDSVAX B/E vaccination induce improved immune responses compared with AIDSVAX B/E vaccination alone

**DOI:** 10.1172/jci.insight.167664

**Published:** 2023-05-08

**Authors:** Margaret C. Costanzo, Dominic Paquin-Proulx, Alexandra Schuetz, Siriwat Akapirat, Zhanna Shubin, Dohoon Kim, Lindsay Wieczorek, Victoria R. Polonis, Hung V. Trinh, Mangala Rao, Hanna Anenia, Michael D. Barrera, Jacob Boeckelman, Barbara Nails, Pallavi Thapa, Michelle Zemil, Carlo Sacdalan, Eugene Kroon, Boot Kaewboon, Somporn Tipsuk, Surat Jongrakthaitae, Sanjay Gurunathan, Faruk Sinangil, Jerome H. Kim, Merlin L. Robb, Julie A. Ake, Robert J. O’Connell, Punnee Pitisutthithum, Sorachai Nitayaphan, Suwat Chariyalertsak, Michael A. Eller, Nittaya Phanuphak, Sandhya Vasan, the RV306, RV328 study groups

**Affiliations:** 1 The US Military HIV Research Program, Walter Reed Army Institute of Research, Silver Spring, Maryland, USA.; 2 Henry M. Jackson Foundation for the Advancement of Military Medicine, Bethesda, Maryland, USA.; 3 Armed Forces Research Institute for Medical Sciences, Bangkok, Thailand.; 4 SEARCH, Institution of HIV Research and Innovation, Bangkok, Thailand.; 5 Sanofi Pasteur, Swiftwater, Pennsylvania, USA.; 6 Global Solutions for Infectious Diseases, South San Francisco, California, USA.; 7 International Vaccine Institute, Seoul, South Korea.; 8 Mahidol University, Bangkok, Thailand.; 9 RIHES, Chiang Mai, Thailand.; 10 The RV306 and RV328 study groups are detailed in Supplemental Acknowledgments.

**Keywords:** AIDS/HIV, AIDS vaccine

## Abstract

The RV144 phase III vaccine trial demonstrated that ALVAC-HIV and AIDSVAX B/E administration over 6 months resulted in 31% efficacy in preventing HIV acquisition, while administration of AIDSVAX B/E alone in both VAX003 and VAX004 studies failed to show efficacy. In this study, we aimed to understand the impact of ALVAC-HIV on the development of cellular, humoral, and functional immune responses compared to the administration of AIDSVAX B/E alone. ALVAC-HIV in combination with 3 doses of AIDSVAX B/E significantly increased CD4^+^ HIV-specific T cell responses, polyfunctionality, and proliferation compared with 3 doses of AIDSVAX B/E alone. Additionally, Env-specific plasmablasts and A244-specific memory B cells were identified with a significantly higher magnitude in the group that received ALVAC-HIV. Subsequently, data revealed increased magnitude of plasma IgG binding to and avidity for HIV Env in participants who received ALVAC-HIV compared with 3 doses of AIDSVAX B/E alone. Lastly, levels of the Fc-mediated effector functions antibody-dependent cellular cytotoxicity, NK cell activation, and trogocytosis were significantly increased in participants who received ALVAC-HIV compared with those receiving AIDSVAX B/E alone. Taken together, these results suggest that ALVAC-HIV plays an essential role in developing cellular and humoral immune responses to protein-boosted regimens relative to protein alone.

## Introduction

After more than 30 years of clinical testing, a highly efficacious HIV vaccine regimen remains elusive. With many different platforms and regimens tested, 2 of the first HIV, phase IIb/III, vaccine efficacy studies conducted were VAX003 (ClinicalTrials.gov NCT00006327) and VAX004 (ClinicalTrials.gov NCT00002441) (1–4). Both trials targeted populations with a high risk of acquiring HIV infection, although the routes of exposure in each population differed. VAX003 targeted injection drug users, and VAX004 targeting men who have sex with men, bisexual men, and women who were at high risk of acquiring HIV through sexual contact. The VAX003 vaccine tested was AIDSVAX B/E, which consists of subtype B MN recombinant gp120 (rgp120) plus subtype E (A244 CRF01 AE) rgp120 adjuvanted with aluminum hydroxide, while VAX004 tested AIDSVAX B/B, containing 2 different subtype B gp120 proteins: MN and GNE8 adjuvanted in aluminum hydroxide. VAX003 was conducted in Bangkok, Thailand, while VAX004 was conducted in the United States, Canada, and the Netherlands. Both studies had identical vaccination schedules. Unfortunately, the efficacy results of both VAX003 and VAX004 were dismal, with protective efficacy of 0.1% and 6.0%, respectively. Neither vaccine had a significant effect on viral load, CD4^+^ T cell counts, rates of antiretroviral therapy initiation, or disease progression, although both resulted in development of antibodies against gp120 components. In VAX004, the peak antibody responses were significantly inversely correlated with the incidence of HIV (5).

Following those trials, the RV144 phase III trial has since been the only vaccine trial to demonstrate overall efficacy in preventing HIV infection (6). The RV144 trial administered an ALVAC-HIV (vCP1521) prime on weeks 0, 4, 12, and 24, with an AIDSVAX B/E protein boost on weeks 12 and 24 or placebo for both to over 16,000 volunteers at risk for HIV infection at a 1:1 ratio. In a post hoc analysis, efficacy was 60% at 12 months but declined to 31% after 3 years, indicating that protective immunity may have waned rapidly (6). Subsequently, the RV306 trial (ClinicalTrials.gov NCT01931358), which administered the RV144 regimen with additional boosting at varying intervals, showed improvements in quality, magnitude, and duration of cellular, humoral, and mucosal responses (7). Collectively, these results prompted us to investigate the role that ALVAC-HIV played in the improved immune responses to a protein-based candidate vaccine. The phase I RV328 clinical trial (ClinicalTrials.gov NCT01933685) evaluated immune responses to administration of AIDSVAX B/E alone to healthy Thai volunteers, enabling this comparative study. Therefore, we performed a wide comparative characterization of both cellular and humoral responses, including assessment of the magnitude and polyfunctionality of T cells, Env-specific B cells, and neutralization and Fc-mediated effector functions of antibodies, between RV306 and RV328 study participants. Overall, including ALVAC-HIV in the vaccine regimen in RV306 led to higher and more polyfunctional CD4^+^ T cell responses as well as higher humoral responses.

## Results

**Figure 1:**
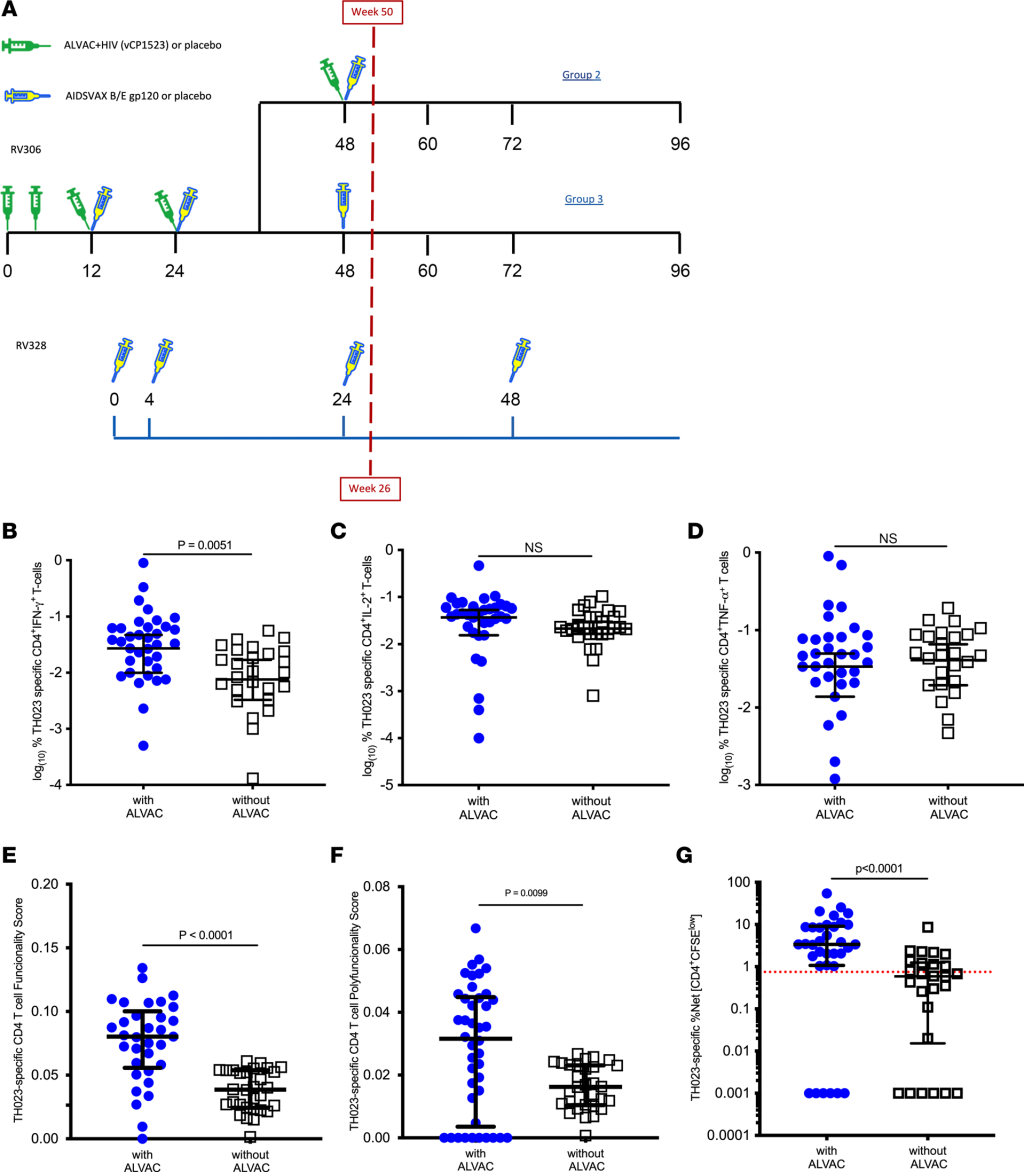
RV306 and RV328 study design. (**A**) Each RV306 participant received ALVAC-HIV and AIDSVAX B/E either alone or in combination at the indicated time points. RV328 participants received AIDSVAX B/E alone at the indicated time points. Comparative analysis was performed on week 50 for RV306 and week 26 for RV328, coinciding with 3 AIDSVAX B/E protein administrations within each study. Univariate analysis of TH023-specific CD4^+^ T cells expressing IFN-γ (**B**), IL-2 (**C**), and TNF-α (**D**). COMPASS revealed significantly higher functionality (**E**) and polyfunctionality (**F**) scores as well as increased proliferation (**G**) in CD4^+^ TH023 T cell responses in participants who received ALVAC-HIV. The dotted red line in **G** indicates the cutoff for positivity. For functionality and polyfunctionality scores, *n* = 33 for participants receiving ALVAC-HIV and AIDSVAX B/E and *n* = 30 for those who received AIDSVAX B/E alone. For CD4^+^ T cell proliferation, *n* = 34 for participants receiving ALVAC-HIV and AIDSVAX B/E and *n* = 26 for those who received AIDSVAX B/E alone. Data are presented as the median and IQR. Statistical significance was assessed using the Mann-Whitney *U* test.

Although the vaccine regimens differ between the 2 studies, the comparison of time points after 3 administrations of AIDSVAX B/E was chosen due to this specific commonality in each trial, as depicted in Figure 1A. Additionally, in RV306, participants in groups 2 and 3 (who received the identical 4 priming vaccinations and differed only by the inclusion or not of ALVAC-HIV in the last vaccination) were combined due to the fact that there were no differences between the groups in the primary analysis (7).

T cell responses measured in both studies, after stimulation with HIV-1 peptide pools, were primarily CD4^+^ mediated and directed against TH023 Env. Without ALVAC-HIV, marginal CD4^+^ TH023-specific univariate response rates for IFN-γ (3%), IL-2 (23%), and TNF-α (7%) were observed after 3 AIDSVAX B/E vaccinations, with a magnitude of up to 0.07%, 0.13%, and 0.15% of CD4^+^ T cells, respectively (Figure 1, B–D). In addition to low CD4^+^ T cell response rates and magnitude of responses at the univariate level, minimal functionality and polyfunctionality scores were observed after AIDSVAX B/E administration alone (Figure 1, E and F). In contrast, with ALVAC-HIV, CD4^+^ T cell responses were more readily detected, with IFN-γ (34%), IL-2 (39%), or TNF-α (18%) response rates after the primary vaccination scheme with a magnitude ranging up to 0.9%, 0.46%, and 0.9% of CD4^+^ T cells, respectively. The magnitude of the HIV-1–specific IFN-γ response was significantly higher (*P* = 0.0051) in the group that received both ALVAC-HIV and AIDSVAX B/E compared with AIDSVAX B/E alone, while IL-2 and TNF-α expression was not significantly different between the 2 groups (Figure 1, B–D). A multivariate combinatorial polyfunctionality analysis of single cells (COMPASS) showed that TH023 antigen–specific CD4^+^ T cell functionality and polyfunctionality scores were significantly higher with ALVAC-HIV than without ALVAC-HIV (Figure 1, E and F) and comparable to T cell responses seen in RV144 (8). Env-specific (TH023) CD4^+^ polyfunctional T cells were characterized by the expression of CD154 (CD40L), IFN-γ, TNF-α, and IL-2 in participants who received ALVAC (Supplemental Figure 1; supplemental material available online with this article; https://doi.org/10.1172/jci.insight.167664DS1). Participants who did not receive ALVAC lacked expression of CD154. Similarly, using a 5-6-carboxyfluorescein diacetate succinimidyl ester–based (CFSE-based) proliferation assay, TH023-specific CD4^+^ T cell proliferation was detected in 82% of RV306 participants who received ALVAC-HIV, while only 39% of RV328 participants receiving AIDSVAX B/E alone showed proliferation. The median magnitude of proliferation was 3.4% and 0.6%, respectively (Figure 1G).

**Figure 2:**
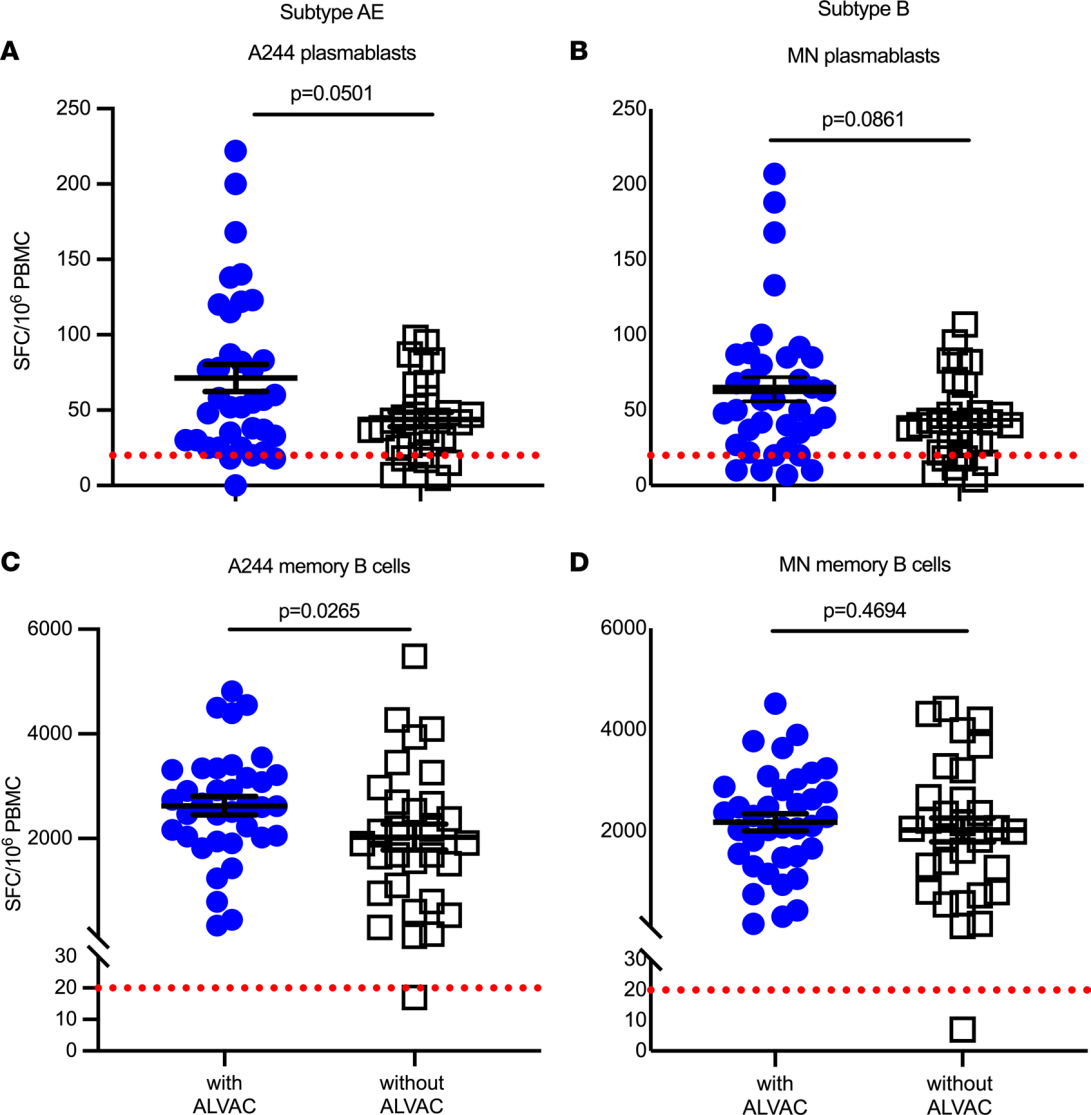
Prevalence of HIV Env–specific antibody-secreting plasmablasts and memory B cells. Env-specific IgG-secreting plasmablasts (**A** and **B**) and memory B cells (**C** and **D**) in PBMCs were enumerated by a direct ELISpot assay for gp120 A244 (**A** and **C**) and MN (**B** and **D**). *n* = 36 for participants receiving ALVAC-HIV and AIDSVAX B/E and *n* = 30 for those who received AIDSVAX B/E alone. Dotted red lines indicate the cutoff for positivity. SFC, spot-forming cells. Data are presented as the mean ± SEM. Statistical significance was assessed using Mann-Whitney *U* test.

Next, a B cell ELISpot assay was performed in an effort to characterize the number of HIV-1–specific B cells secreting IgG. A244 and MN HIV protein–specific plasmablasts trended toward higher levels in the ALVAC-HIV group, with *P* values of 0.0501 and 0.0861, respectively (Figure 2, A and B). Interestingly, A244-specific memory B cells were significantly higher in the ALVAC-HIV group (*P* = 0.0265) compared with the AIDSVAX B/E–alone group (Figure 2C). MN-specific memory B cell responses were not significantly different between the 2 groups (Figure 2D). Additionally, A244- and MN-specific memory B cells had overall higher frequencies in peripheral blood compared with the frequency of plasmablasts for both groups (Figure 2).

**Figure 3:**
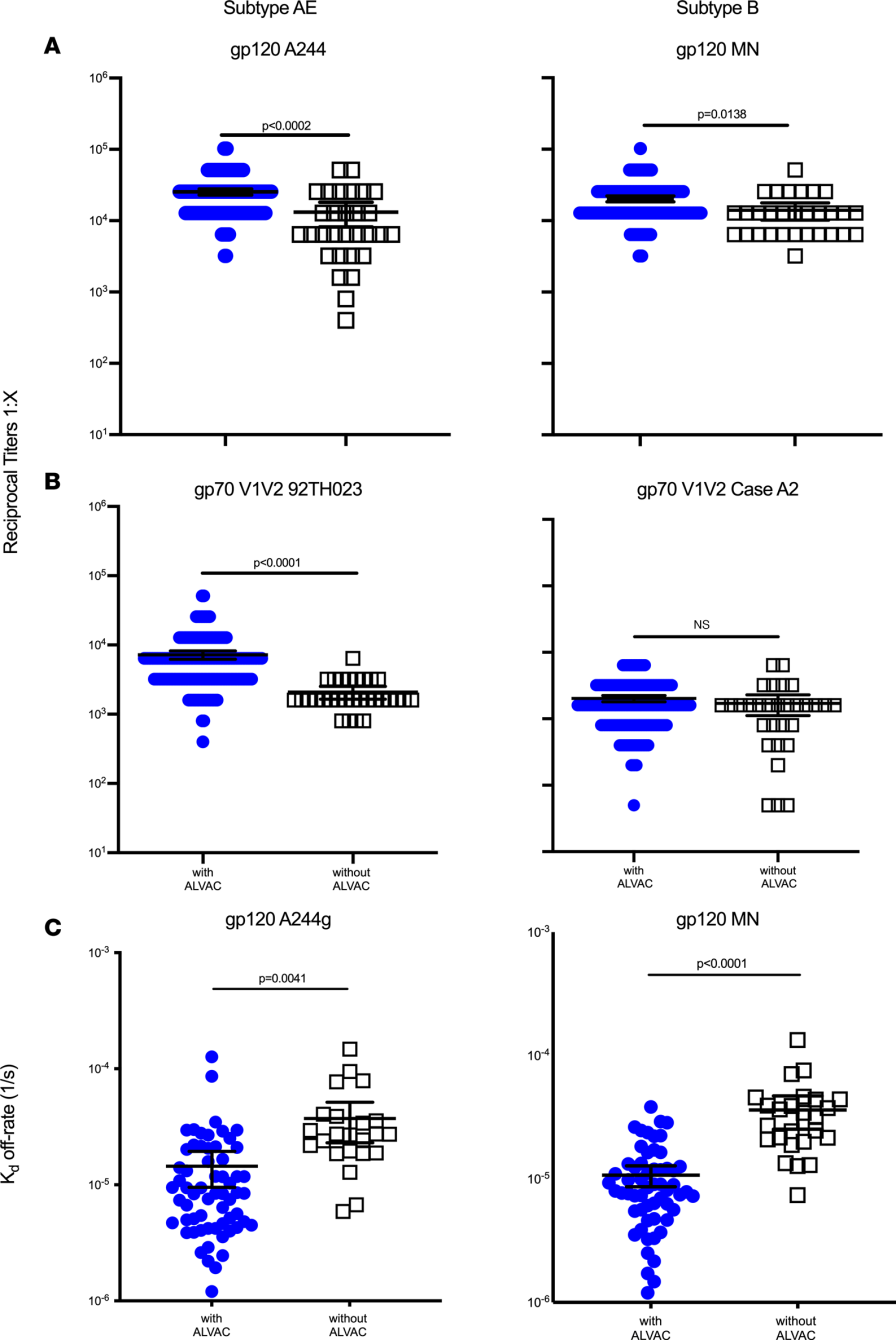
Plasma IgG HIV-1 Env and V1V2 antibody binding titers and avidity of antibody-antigen interaction are significantly higher with ALVAC-HIV. (**A** and **B**) IgG antibody binding titers for HIV-1 gp120 and scaffolded gp70 V1V2 antigens were measured by ELISA. Each symbol represents the average of triplicate measurements for a participant. Geometric mean antibody titers against gp120 A244gD– D11 (**A**, left), gp120 MNgD– D11 (**A**, right), gp70 V1V2 (92TH023) (**B**, left), and gp70 V1V2 (case A2) (**B**, right) are shown. Each panel graphically displays geometric mean titers, color-coded by group. Error bars depict 95% CIs. Statistical significance was assessed using the Mann-Whitney *U* test with step-down Bonferroni adjustment. *n* = 190 for participants receiving ALVAC-HIV and AIDSVAX B/E and *n* = 30 for those who received AIDSVAX B/E alone. (**C**) *K_d_* off-rate (1/s). A lower *K_d_* off-rate corresponds to higher avidity. Statistical analysis was performed using the rank-based nonparametric 1-way ANOVA (Kruskal-Wallis *H* test). The FDR was determined using the 2-stage step-up method of Benjamini, Krieger, and Yekutieli, with confidence level at 0.05. For *K_d_* off-rate with A244, *n* = 62 for participants receiving ALVAC-HIV and AIDSVAX B/E and *n* = 23 for those who received AIDSVAX B/E alone. For *K_d_* off-rate with MN, *n* = 58 for participants receiving ALVAC-HIV and AIDSVAX B/E and *n* = 25 for those who received AIDSVAX B/E alone. Data are shown as the mean and 95% CI.

Plasma IgG antibody binding titers for HIV-1 Env gp120 (A244gD– D11 and MNgD– D11) and V1V2 (gp70V1V2 92TH023 and gp70V1V2 Case A2) antigens were measured by ELISA and expressed as geometric mean endpoint titers (Figure 3, A and B). Participants who received ALVAC-HIV had significantly elevated plasma IgG binding antibody titers against A244 and MN gp120 proteins (Figure 3A) and gp70 V1V2 92TH023 (Figure 3B) compared with those who received AIDSVAX B/E alone. Plasma IgG antibody avidity was then measured via surface plasmon resonance (Biacore) and expressed as *K_d_* off-rate (1/s). In participants who received ALVAC-HIV, the *K_d_* off-rate (1/s) was lower than for those who received AIDSVAX B/E alone, indicating higher avidity for both gp120 A244 and MN (Figure 3C).

**Figure 4:**
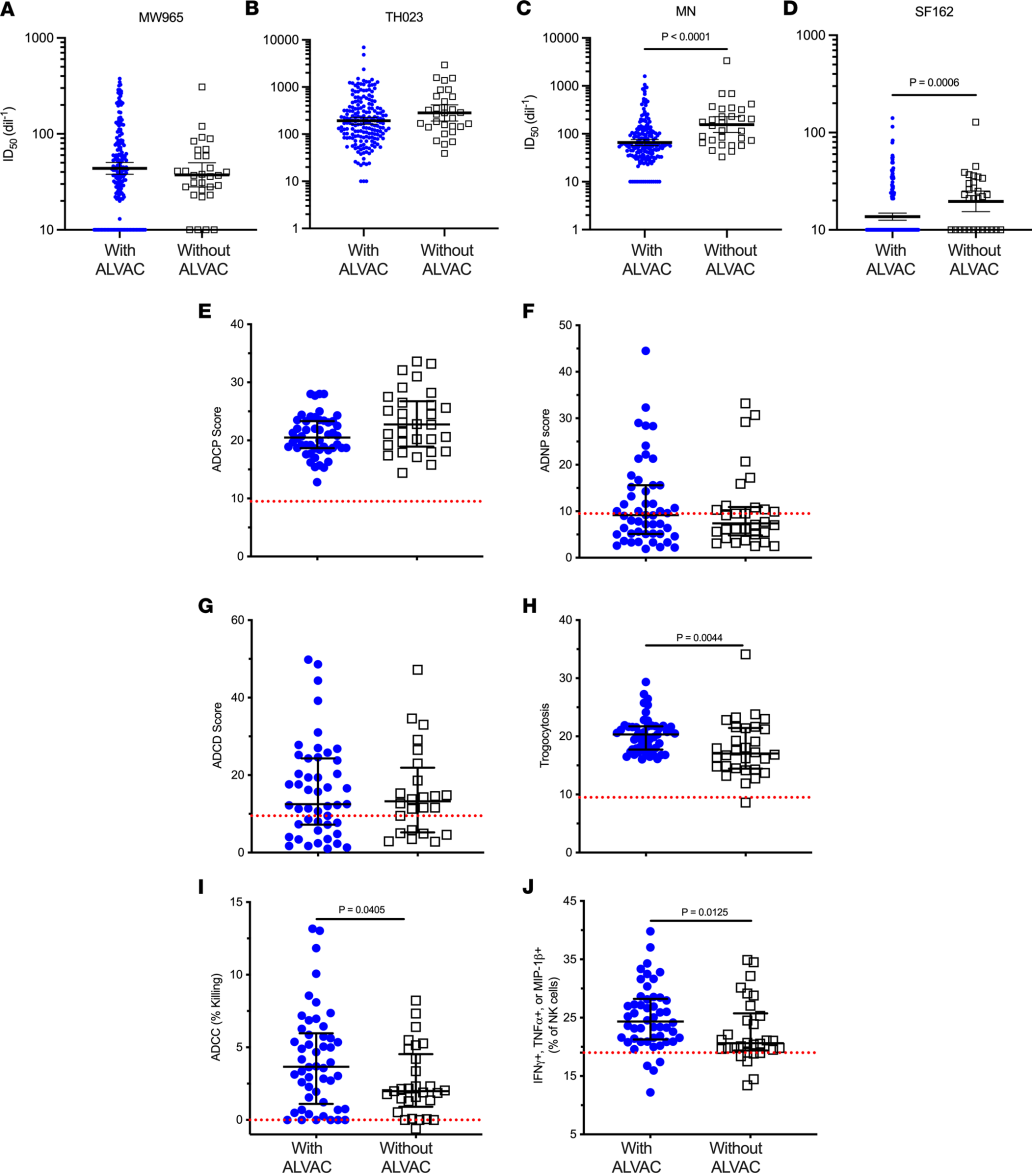
TZM-bl neutralizing antibody levels and antibody effector function levels after 3 AIDSVAX B/E vaccinations in participants who received ALVAC-HIV versus participants who received AIDSVAX B/E alone. ID_50_ against MW965 (**A**), TH023 (**B**), MN (**C**), and SF162 (**D**) PSVs, with and without ALVAC-HIV. Each panel graphically depicts ID_50_, color coded by group (blue, with ALVAC; black, without ALVAC). Error bars depict 95% CIs. Statistical significance was assessed using the Mann-Whitney *U* test. *n* = 190 for participants receiving ALVAC-HIV and AIDSVAX B/E and *n* = 30 for those who received AIDSVAX B/E alone. Plasma antibody effector activity is shown for ADCP (**E**), ADNP (**F**), ADCD (**G**), trogocytosis (**H**), ADCC (**I**), and NK cell activation, as measured by production of IFN-γ, TNF-α, or MIP-1β (**J**) for participants receiving ALVAC-HIV and AIDSVAX B/E (*n* = 50) and those who received AIDSVAX B/E alone (*n* = 30). Dotted red lines indicate the cutoff for positivity. Data are presented as the median and IQR. Statistical significance determined by unpaired, nonparametric Mann-Whitney *U* test.

Next, we measured plasma neutralizing antibodies using the TZM-bl assay against a panel of tier 1 pseudoviruses (PSVs). There was no significant difference in neutralization titers between the groups against MW965 and TH023 PSVs (Figure 4, A and B). Neutralization titers against MN and SF162 PSVs were lower in participants who received ALVAC-HIV compared with participants who did not receive ALVAC-HIV (Figure 4, C and D). We also investigated the effect of ALVAC-HIV on the generation of Fc-mediated antibody effector functions. No significant differences were seen in antibody-dependent cellular phagocytosis (ADCP), antibody-dependent neutrophil phagocytosis (ADNP), or antibody-dependent complement deposition (ADCD) between participants who received ALVAC-HIV or not (Figure 4, E–G). However, participants who received ALVAC-HIV had significantly higher levels of antibodies mediating trogocytosis (*P* = 0.004), antibody-dependent cellular cytotoxicity (ADCC) (*P* = 0.04), and NK cell activation (*P* = 0.01), as measured by IFN-γ, TNF-α, or MIP-1β production, compared with participants who received AIDSVAX B/E alone (Figure 4, H–J). Collectively, these results suggest that ALVAC-HIV contributed to the development of functional antibodies that might have mediated protective responses seen in RV144 (9).

**Figure 5:**
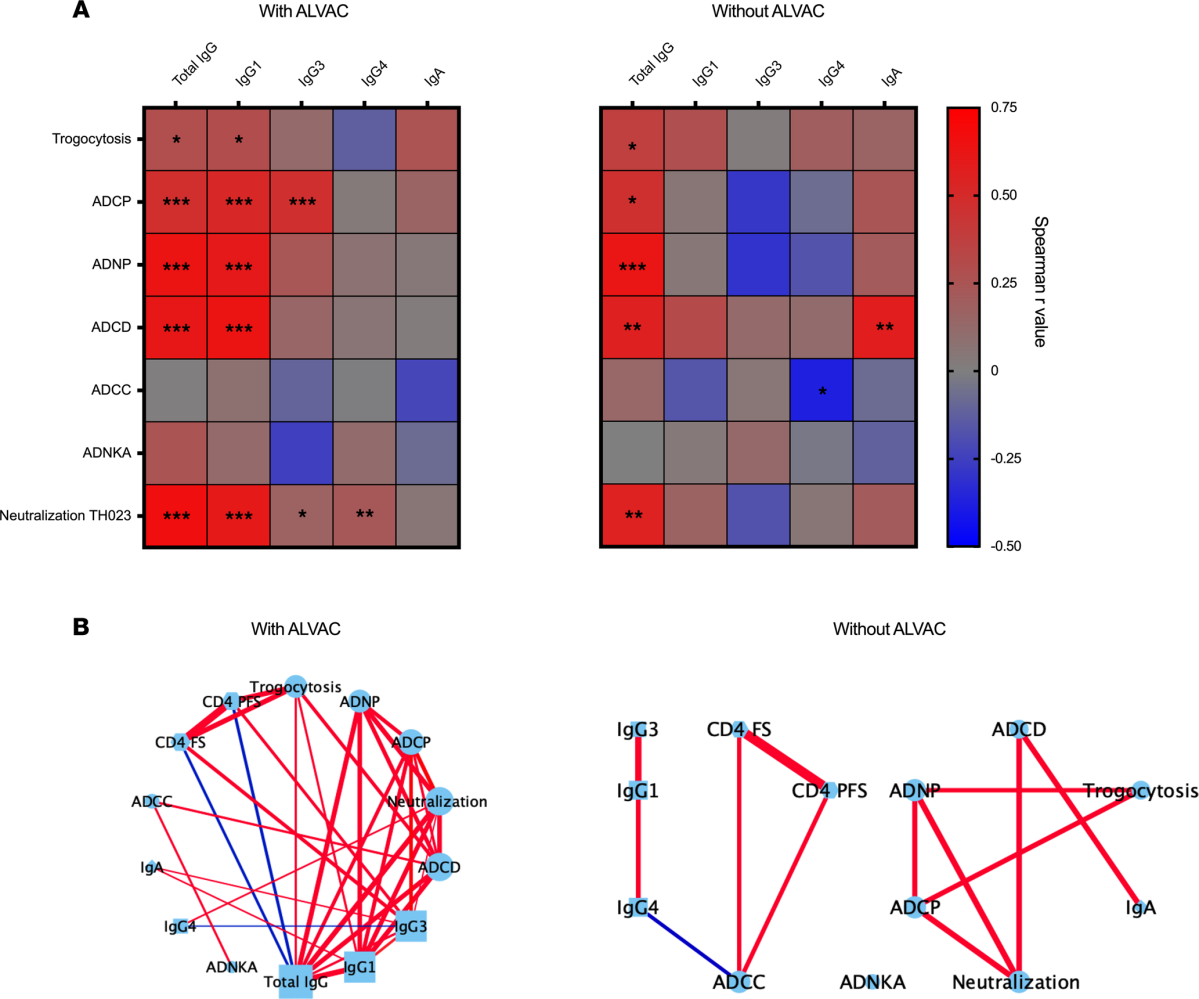
Network analysis. (**A**) Associations between antibody features were determined using nonparametric Spearman’s correlation coefficient. *P < 0.05, **P < 0.01, ***P < 0.001. (**B**) Antibody binding (squares, IgG; triangles, IgA) and functions (circles) as well as T cell polyfunctionality (hexagons) were included in network analysis. Statistically significant associations (*P* < 0.05) were used to generate networks in Cytoscape (version 3.7.2). Positive associations are shown in red and negative associations are shown in blue. The thickness of the lines represents the strength of the association (*r* values). In participants who received ALVAC-HIV, Fc functions vs. binding and neutralization used *n* = 50, CD4^+^ cell functions vs. binding neutralization used *n* = 35, CD4^+^ T cell responses vs. Fc function used *n* = 16. In participants who received AIDSVAX B/E alone, all network and association comparisons used *n* = 30. ADNKA, antibody-dependent NK cell activation.

Lastly, associations between antibody features were determined using nonparametric Spearman’s correlation coefficient. With ALVAC-HIV, ADCP, ADNP, ADCD, and neutralization titers of TH023 PSVs had significant associations with total IgG as well as IgG1 and IgG3 subclass titers (*r* = 0.5 to 0.75) (Figure 5A). A network analysis was then performed that included all antibody functions and COMPASS T cell functionality and polyfunctionality scores. This analysis revealed strong associations with T cell polyfunctionality, 4 antibody effector functions, and TH023 neutralization titer in participants who received ALVAC-HIV (Figure 5B). In participants who received AIDSVAX B/E alone, T cell polyfunctionality was only associated with ADCC and a separate network emerged with 4 antibody effector functions, TH023 neutralization titers, and binding to IgA.

## Discussion

In the RV144 clinical trial, the prime-boost vaccine regimen consisted of a nonreplicating recombinant canarypox vector, ALVAC-HIV prime (vCP1521) and AIDSVAX gp120 B/E protein boost. RV144 demonstrated modest efficacy, while VAX003 and VAX004, which used AIDSVAX B/B and B/E alone, did not. There may be multifactorial reasons for this discrepancy, such as differences in risk groups, immunization regimens, immunogens, and antibody responses (10). Recombinant viral vectors expressing inserted immunogens, such as recombinant canarypox viruses, provide a means of mimicking viral infection and eliciting both humoral and cell-mediated immunity. Viral vectors can also be sensed by the immune system and stimulate cytokine production. In the case of ALVAC-HIV, activation of the cGAS/IFI16/STING/type I IFN pathway leads to activation of the inflammasome, which has been associated with reduced risk of simian immunodeficiency virus (SIV) infection (11, 12). ALVAC-HIV also leads to activation of the transcription factor CREB1 that has been linked to better recruitment of immune cells to the site of antigen presentation (13). For this reason, we chose to comprehensively investigate and compare humoral and cell-mediated responses elicited by the ALVAC-HIV and AIDSVAX B/E late-boosting regimen (RV306) and AIDSVAX B/E alone (RV328) to better understand the role of ALVAC-HIV.

In participants who received ALVAC-HIV, COMPASS showed induction of polyfunctional CD4^+^ T cell subsets that had similar cytokine profiles as seen in RV144, which was associated with decreased risk of HIV infection (8). Specifically, these subsets were characterized by the expression of CD154 (CD40L) and IL-2, which are important for CD4^+^ T cell–B cell interactions (Supplemental Figure 1). Therefore, CD4^+^ T cell subsets may have contributed to T cell help for antibody production. In addition, CD4^+^ T cell proliferation was superior with ALVAC-HIV versus without. These data suggest that ALVAC-HIV helped shape the CD4^+^ T cell response and resulted in increased, highly polyfunctional CD4^+^ T cells and gp120-specific plasmablasts and memory B cells. In combination, these mechanisms of responses likely contributed to the T cell–B cell interaction, positively impacting downstream antibody function and overall humoral responses.

Humoral responses indicated that ALVAC-HIV in combination with AIDSVAX B/E boosts increased the magnitude and affinity of binding plasma IgG compared with AIDSVAX B/E alone. Similarly, previous work comparing the magnitude of binding antibody between RV144, VAX003, and VAX004 after 2 protein immunizations also reported higher binding antibody against some, but not all antigens, in RV144 (10). Furthermore, repeated protein immunization in VAX003 and VAX004 changed the IgG subclass distribution, with decreasing IgG3 and increasing IgG4. In addition, the ALVAC-HIV with AIDSVAX B/E boost combination had significantly higher levels of antibodies mediating ADCC, trogocytosis, and NK cell activation. It has been shown that NK cell activation and ADCC, elicited by an ALVAC-SIV–based regimen, correlated with decreased and delayed risk of virus acquisition, respectively, in a nonhuman primate study (14). These results suggest that ALVAC-HIV contributed to the development of functional antibodies that could have mediated protective responses seen in RV144.

Despite promoting higher HIV-specific memory B cells, binding antibodies, avidity, and engagement of some Fc effector functions, participants who received ALVAC-HIV had lower neutralizing titers against 2 tier 1 subtype B PSVs. This is consistent with our previous report showing that the RV144 vaccine regimen limited the development of broadly neutralizing antibodies after breakthrough infections (15). One possible explanation is that ALVAC-HIV vaccination shifts the humoral immune response toward non-neutralizing epitopes. Another possibility is that the difference in timing of the AIDSVAX B/E administration could contribute to the difference in neutralizing titers.

One limitation of the current study is that study participants who received ALVAC-HIV received more vaccinations (total of 5) than those without (total of 3). Furthermore, RV306 participants received ALVAC-HIV both as a prime and together with AIDSVAX B/E boosts. Previous work on the RV305 and RV306 trials suggests that ALVAC-HIV has minimal impact when included in the late boost (7, 16), thus suggesting that the main contribution of ALVAC-HIV might be during the priming phase.

In conclusion, the viral vector ALVAC-HIV (vCP1452) together with protein AIDSVAX B/E boosts significantly increased humoral and cell-mediated responses compared with administration of protein AIDSVAX B/E vaccinations alone. This comparative study indicates that viral vector priming may be essential for activating multiple arms of the immune system and generating durable responses when combined with protein boosts. These combined cellular and humoral interactions may be required for protection against more complex pathogens such as HIV.

## Methods

### Study design and participants.

The RV306 clinical trial (ClinicalTrials.gov NCT01931358) was conducted in healthy Thai volunteers as previously described (7). In RV328 (ClinicalTrials.gov NCT01933685), healthy, HIV-uninfected volunteers, at low risk for HIV infection, between 20 and 40 years of age available for follow-up for 18 months were enrolled. A total of 40 volunteers were enrolled, with 30 vaccine and 10 placebo recipients. AIDSVAX B/E or placebo was administered on weeks 0, 4, 24, and 48. Volunteers were followed up until week 74 after enrollment. Contemporary assays were performed on samples obtained from study participants on weeks, 4, 6, 24, 26, 48, 50, and 72. AIDSVAX B/E, manufactured by Genentech Inc. for Global Solutions for Infectious Diseases (GSID), is a bivalent HIV gp120 glycoprotein vaccine with subtype B (MN) and subtype E (A244) proteins absorbed onto a total of 0.6 mg aluminum hydroxide gel at a combined concentration of 600 μg/mL (300 μg of each antigen) given as a 1 mL intramuscular injection into the deltoid muscle. AIDSVAX B/E Placebo, manufactured by Hollister-Stier Laboratories LLC for GSID, is a sterile suspension of 600 μg of aluminum hydroxide adjuvant, given as a 1 mL intramuscular injection into the deltoid muscle.

### Randomization and masking.

Randomization for vaccine/placebo was 3:1 in both trials. The PI, study staff, and volunteers were blinded as to receipt of active vaccine or placebo. The pharmacy staff preparing the study injections were not involved in the clinical assessment of participants. For all participants, the volume of injection was consistent.

### Procedures.

RV306 procedures were previously described (7). In both RV306 and RV328, T cell responses were measured by intracellular cytokine staining (ICS) as previously described (17), and functionality scores and polyfunctionality scores were calculated via COMPASS (8). Antigen-specific cellular proliferation was assessed by quantification of CFSE^lo^ CD4^+^ and CD8^+^ T cells and central memory (CD27^+^CD45RO^+^) and effector memory (CD27^–^CD45RO^+^) T cells. Env-specific IgG–secreting plasmablasts and memory B cells in peripheral blood mononuclear cells (PBMCs) were enumerated by a direct enzyme-linked immunospot (ELISpot) assay. HIV-1–specific plasma IgG and IgA ELISA antibody responses were assessed using rgp120 and scaffold proteins. Capture antigens included V1V2 sequences from both subtype AE and subtype B HIV-1 Env (gp70 V1V2 92TH023 and gp70 V1V2 Case A2), and HIV-1 Env gp120 proteins matched to sequences in AIDSVAX B/E without the gD tag but with an 11–amino acid N-terminal deletion, represented as gp120 A244gD– D11 and gp120 MNgD– D11. Neutralizing antibodies were measured by a PSV neutralization assay using TZM-bl cells. Tier 1 neutralization was assessed using a panel of 7 PSVs. Antibody avidity was determined by surface plasmon resonance using a Biacore 4000 as previously described (18, 19). Innate effector function assays, including ADCC, ADCP, ADNP, ADCD, trogocytosis, and NK cell activation were performed as previously described (20, 21). Detailed methods for immunoassays are available in the supplemental material.

### Statistics.

The RV328 study enrolled a total of 40 individuals randomized to 30 vaccine recipients and 10 placebo recipients, thus permitting detection of large differences in response rates between the active regimens (>50 percentage points) with adequate power (80%) across the expected response range for selected assays (i.e., ICS with p1 = 0.30, p2 = 0.80). In addition, differences in mean assay levels of approximately 1.1 standard deviations for the comparison of the 2 regimens were detectable (with power = 80%). COMPASS posterior probabilities for Env-specific (TH023) CD4^+^ T cells from RV306 and RV328 were calculated, resulting in polyfunctionality and functionality scores, which were compared between the 2 groups using a Mann-Whitney *U* test. Plasmablasts and memory B cell ELISpot data were compared using the Mann-Whitney *U* test. Binding antibody data were compared using an unpaired, 2-tailed *t* test. Avidity data were compared using 1-way ANOVA (Kruskal-Wallis test). Neutralization data and antibody effector function data were compared using the Mann-Whitney *U* test. Network analysis associations were determined using nonparametric Spearman’s correlation coefficient. Statistically significant associations (*P* < 0.05) were used to generate networks in Cytoscape version 3.7.2 (https://cytoscape.org/).

### Study approval.

The RV306 study was approved by ethical review boards at the Walter Reed Army Institute of Research, Thai Ministry of Public Health, Royal Thai Army Medical Department, Faculty of Tropical Medicine, Mahidol University, Chiang Mai University, and Chulalongkorn University Faculty of Medicine. The RV328 study was approved by ethical review boards at the Walter Reed Army Institute of Research and the Chulalongkorn University Faculty of Medicine. This study was conducted in accordance with Good Participatory Practice principles (22). All study participants provided written informed consent.

### Role of the funding source.

The funders of this study had a role in study design and data analysis but no role in data collection, data interpretation, or writing of the report. The corresponding author had full access to all the data in the study and had final responsibility for the decision to submit for publication.

### Data availability.

Study protocol and informed consent documents are available online. Deidentified participant-level data and accompanying research resources are available upon request to pubrequest@hivresearch.org. Distribution of data will require compliance with all applicable regulatory and ethical processes.

## Author contributions

JHK, MLR, NP, PP, SN, EK, FS, SG, RJO, and SV designed the studies. NP, PP, SN, SC, EK, ST, and CS conducted the clinical studies. SA performed the binding antibody assays. HVT and MR performed the antibody avidity assays. LW, MZ, and VRP conducted the neutralization assays. MAE, DK, MCC, HA, MDB, JB, and PT performed intracellular cytokine assays and combinatorial polyfunctionality analysis of antigen-specific T cell subset analyses. AS and SJ carried out the CFSE assays. AS and BK performed the ELISpot assays. DPP, BN, and ZS carried out the antibody effector function assays. DPP conducted the network analysis. SA, HVT, LW, AS, DPP, MCC, and SV analyzed data. SV, MCC, DPP, and MR wrote the manuscript. NP, JHK, MLR, JAA, MAE, AS, SA, VP, PP, SN, SC, EK, and CS reviewed and edited the manuscript.

## Supplementary Material

Supplemental data

## Version 1 05/08/2023

Electronic publication

## References

[1] Punnee Pitisuttithum PG (2006). Randomized, double-blind, placebo-controlled efficacy trial of a bivalent recombinantv glycoproteinv 120 HIV-1 vaccine among injection drug users in Bangkok, Thailand. J Infect Dis.

[2] Vanichseni ST (2004). Recruitment, screening and characteristics of injection drug users participating in the AIDSVAX B/E HIV vaccine trial, Bangkok, Thailand. AIDS.

[3] Harro CD (2004). Recruitment and baseline epidemiologic profile of participants in the first phase 3 HIV vaccine efficacy trial. J Acquir ImmuneDefic Syndr.

[4] Flynn NM (2005). Placebo-controlled phase 3 trial of a recombinant glycoprotein 120 vaccine to prevent HIV-1 infection. J Infect Dis.

[5] Gilbert PB (2005). Correlation between immunologic responses to a recombinant glycoprotein 120 vaccine and incidence of HIV-1 infection in a phase 3 HIV-1 preventive vaccine trial. J Infect Dis.

[6] Rerks-Ngarm S (2009). Vaccination with ALVAC and AIDSVAX to prevent HIV-1 infection in Thailand. N Engl J Med.

[7] Pitisuttithum P (2020). Late boosting of the RV144 regimen with AIDSVAX B/E and ALVAC-HIV in HIV-uninfected Thai volunteers: a double-blind, randomised controlled trial. Lancet HIV.

[8] Lin L (2015). COMPASS identifies T-cell subsets correlated with clinical outcomes. Nat Biotechnol.

[9] Haynes BF (2012). Immune-correlates analysis of an HIV-1 vaccine efficacy trial. N Engl J Med.

[10] Karnasuta C (2017). Comparison of antibody responses induced by RV144, VAX003, and VAX004 vaccination regimens. AIDS Res Hum Retroviruses.

[11] Liu F (2017). Priming and activation of inflammasome by canarypox virus vector ALVAC via the cGAS/IFI16-STING-type I IFN pathway and AIM2 sensor. J Immunol.

[12] Vaccari M (2018). HIV vaccine candidate activation of hypoxia and the inflammasome in CD14^+^ monocytes is associated with a decreased risk of SIV_mac251_ acquisition. Nat Med.

[13] Tomalka JA (2021). The transcription factor CREB1 is a mechanistic driver of immunogenicity and reduced HIV-1 acquisition following ALVAC vaccination. Nat Immunol.

[14] Gorini G (2020). Engagement of monocytes, NK cells, and CD4^+^ Th1 cells by ALVAC-SIV vaccination results in a decreased risk of SIVmac251 vaginal acquisition. PLoS Pathog.

[15] Mdluli T (2020). RV144 HIV-1 vaccination impacts post-infection antibody responses. PLoS Pathog.

[16] Fischinger S (2020). Protein-based, but not viral vector alone, HIV vaccine boosting drives an IgG1-biased polyfunctional humoral immune response. JCI Insight.

[17] Horton H (2007). Optimization and validation of an 8-color intracellular cytokine staining (ICS) assay to quantify antigen-specific T cells induced by vaccination. J Immunol Methods.

[18] Scaria PV (2021). Malaria transmission-blocking conjugate vaccine in ALFQ adjuvant induces durable functional immune responses in rhesus macaques. NPJ Vaccines.

[19] Verma A (2020). Impact of T_h_1 CD4 follicular helper T cell skewing on antibody responses to an HIV-1 vaccine in rhesus macaques. J Virol.

[20] Paquin-Proulx D (2021). Associations between antibody Fc-mediated effector functions and long-term sequelae in Ebola virus survivors. Front Immunol.

[21] Alrubayyi A (2018). A flow cytometry based assay that simultaneously measures cytotoxicity and monocyte mediated antibody dependent effector activity. J Immunol Methods.

[22] https://www.unaids.org/sites/default/files/media_asset/JC1853_GPP_Guidelines_2011_en_0.pdf.

