# Supplementary material for: ALVAC-HIV and AIDSVAX B/E vaccination induce improved immune responses compared with AIDSVAX B/E vaccination alone
Source: JCI Insight. 2023 May 8;8(9):e167664. doi: 10.1172/jci.insight.167664 (PMC10243797; doi:10.1172/jci.insight.167664)
Supplement: Supplemental data [file jciinsight-8-167664-s052.pdf]

Appendix

Table of Contents

RV306 Study Group ... 2

RV328 Study Group ... 2

Detailed Materials and Methods ... 3

Antibody binding ELISA assays ... 3

Antibody avidity assays: ... 3

Antibody-secreting cells (plasmablasts) and memory B cell assay: ... 4

Antigen-specific cellular proliferation ... 5

High-throughput pseudovirus (PSV) neutralization assay ... 6

Fc-mediated effector function assays ... 6

Cell lines and primary cells ... 6

Protein coupling to fluorescent beads: ... 6

Antibody-Dependent Cellular Phagocytosis (ADCP): ... 6

Antibody-Dependent Neutrophil Phagocytosis (ADNP): ... 7

Antibody-Dependent Complement Deposition (ADCD): ... 7

Antibody-mediated Natural Killer (NK) cell activation: ... 7

Antibody-Dependent Cellular Cytotoxicity (ADCC): ... 8

Trogocytosis: ... 8

Negative and Positive Control Plasma: ... 8

Flow Cytometry: ... 9

Intracellular cytokine staining: ... 9

COMPASS analysis: ... 10

Supplemental Figure 1. Heatmap of COMPASS posterior probabilities for Env-specific (TH023) CD4+ T-cells from participants that received ALVAC or not. ... 11

References ... 12

Supplemental Acknowledgments

RV306 Study Group

Mahidol University: Arom Pitisuthitham, Yupa Sabmee

RTA-AFRIMS: Narongrid Sirisopana, Chirapa Eamsila, Prapaporn Savaraj, Wanlaya Labwech, Siriluck Teerachia

Research Institute for Health Sciences (RIHES), Chiang Mai University: Nuntisa Chotirosniramit, Taweewat Supindham, Boonlure Pruenglampoo, Patcharaphan Sugandhavesa, Natthapol Kosashunhanan, Oranitcha Kaewthip, Piyathida Sroysuwan, Somporn Tipsuk

BIOPHICS, Mahidol University: Pawinee Jarujareet

US Military HIV Research Program: Silvia Ratto-Kim, Sebastian Molnar, Jesse Schoen

USAMD-AFRIMS: Nampueng Churikanont, Saowanit Getchalarat, Nongluck Sangnoi, Bessara Nuntapinit, Anant Phramtong, Pornsuk V. Grandin, Sirinan Madnote, Surawach Rittiroongrad, Boot Kaewboon, Rapee Trichavaroj, Jiraporn Puangkaew, Somsak Chantakulkij, Phiromrat Rakyat, Pornchanok Panjapornsuk, Nipattra Tragonlugsana, Weerawan Chuenarom, Mark de Souza, Viseth Ngauy

Thai Red Cross AIDS Research Centre: Nittaya Phanuphak, Nitiya Chomchey, Puttachard Saengtawan, Nipat Teeratakulpisarn, Eugene Kroon

Faculty of Medicine, Chulalongkorn Hospital: Rungsun Rerknimitr

Global Solutions for Infectious Diseases: Carter A. Lee

United States Army Medical Materiel Development Activity (USAMMDA): Suchada Chinaworapong

RV328 Study Group

Mahidol University: Arom Pitisuthitham, Yupa Sabmee

RTA-AFRIMS: Narongrid Sirisopana, Chirapa Eamsila, Prapaporn Savaraj, Wanlaya Labwech, Siriluck Teerachia

Research Institute for Health Sciences (RIHES), Chiang Mai University: Nuntisa Chotirosniramit, Taweewat Supindham, Boonlure Pruenglampoo, Patcharaphan Sugandhavesa, Natthapol Kosashunhanan, Oranitcha Kaewthip, Piyathida Sroysuwan, Somporn Tipsuk

BIOPHICS, Mahidol University: Pawinee Jarujareet

US Military HIV Research Program: Silvia Ratto-Kim, Sebastian Molnar, Jesse Schoen

USAMD-AFRIMS: Nampueng Churikanont, Saowanit Getchalarat, Nongluck Sangnoi, Bessara Nuntapinit, Anant Phramtong, Pornsuk V. Grandin, Sirinan Madnote, Surawach Rittiroongrad, Boot Kaewboon, Rapee Trichavaroj, Jiraporn Puangkaew, Somsak Chantakulkij, Phiromrat Rakyat, Pornchanok Panjapornsuk, Nipattra Tragonlugsana, Weerawan Chuenarom, Mark de Souza, Viseth Ngauy

Thai Red Cross AIDS Research Centre: Nittaya Phanuphak, Nitiya Chomchey, Puttachard Saengtawan, Nipat Teeratakulpisarn, Eugene Kroon

Faculty of Medicine, Chulalongkorn Hospital: Rungsun Rerknimitr, Phandee Watanaboonyongcharoen

Global Solutions for Infectious Diseases: Carter A. Lee

United States Army Medical Materiel Development Activity (USAMMDA): Suchada Chinaworapong

Detailed Materials and Methods

Antibody binding ELISA assays

Total and specific antibody (IgG and IgA) responses to gp120 and gp70 V1V2 derived from HIV-1 CRF01_AE (A244 and 92TH023) and subtype B (MN and CaseA2) proteins (all provided by Dr. Shelly Krebs, U.S. Military HIV Research Program, Walter Reed Army Institute of Research, Silver Spring, MD, USA) were assessed by ELISA as previously described [1]. Briefly, 96-well U-bottom Immulon 2HB plates (Thermos Scientific, Rochester, NY) were coated with 1 µg/mL of proteins in D-PBS (Sigma-Aldrich, Saint Louis, MO) at 4°C overnight. Plates were washed and serial two-fold dilutions of specimens with initial dilutions were added to wells. After 2-hour incubation at room temperature, plates were washed and color was developed with 1:25,000 dilution of horseradish peroxidase (HRP) conjugated either to goat anti-human IgG (Cat#A80-104P) or goat anti-human IgA (Cat#A80-102P; Bethyl Laboratories, Montgomery, TX) and ABTS ELISA HRP substrate (KPL, Gaithersburg, MD). Plates were read at an absorbance of A405 nm (Spectramax 340 PC ELISA reader, Molecular Devices, Downingtown, PA). Human reference serum (Cat#RS10-110; Bethyl Laboratories, Montgomery, TX) was used as a positive control. Total IgG and IgA antibody specific to goat anti-human IgG-Fc antibody (Cat#A80-104A; Bethyl Laboratories, Montgomery, TX) and goat anti-human IgA antibody (Cat#A80-102P; Bethyl Laboratories, Montgomery, TX) were assessed on all samples following above ELISA procedure.

Antibody avidity assays:

Sample preparation: To deactivate the complements and lipid contents, sera were heated at 56°C for 45 min followed by centrifugation at 16,000 x g at 4°C for 20 min to collect supernatants

for assay analysis. Sample Analysis: The subsequent procedure was conducted in the Surface Plasmon Resonance Biacore 4000 system. The immobilizations were performed in 10 mM Hepes and 150 mM NaCl pH 7.4 using a standard amine coupling kit as described in [2,3]. The CM5-S series chip surface was activated with a 1:1 mixture of 0.4 M 1-ethyl-3-(3-dimethylaminopropyl) carbodiimide hydrochloride (EDC) and 0.1 M N-hydroxysuccinimide (NHS) for 600 s. Then 4 µg/ml A244.gp120 or MN.gp120 protein (provided by Dr. Shelly Krebs, U.S. Military HIV Research Program, Walter Reed Army Institute of Research, Silver Spring, MD, USA) in 10 mM sodium acetate pH 4.5 was immobilized to spot 1, 2, 4 and 5 to each flow cell of the CM5 sensor chip. The density was immobilized in the range of 2,600 – 4,300 RU for A244.gp120 and 2,200 – 6,800 RU for MN.gp120. The immobilized surface was then deactivated by 1.0 M ethanolamine-HCl pH 8.5 for 600 s. Spot 3 in each flow cell was left unmodified to serve as a reference. The immobilized surface was then deactivated by 1.0 M ethanolamine-HCl pH 8.5 for 600 s. Following the surface preparation, the heat-inactivated plasma samples were diluted 1:50 for gp120 in 10 mM Hepes, 300 mM NaCl, 0.005% Tween-20, pH7.4 running buffer and injected onto the antigen-immobilized surface for 300 s followed by a 30 s dissociation period. To regenerate the bound surface, 150 - 175 mM HCl was injected for 60 s. Four replicates for each peptide were collected at rate of 10 Hz, with an analysis temperature at 25 ^{0}C. All sample injections were conducted at flow rate of 10 µl/min. To determine the stability of immobilized antigens on the surface and variations of the different sensor chips, the certain samples were used to inject repeatedly. Data analysis was performed using Biacore 4000 Evaluation software 4.1 with double subtractions for unmodified surface and buffer for blank. The cut-off for the binding response was ≥ 10 RU. The avidity was filtered with T(kd) ≥ 20 and closeness of the fit (≤ 10%, chi2/Rmax). In addition, individual sensogram was checked manually for the fitness.
Statistical analysis: PRISM v7.0, 1-way ANOVA (Krustal-Wallis test), No matching or pairing, Nonparametric test, False Discovery Rate using two-stage step-up method of Benjamini, Krieger and Yekutieli, Confidence level: 0.05

Antibody-secreting cells (plasmablasts) and memory B cell assay: A direct enzyme-linked immunospot (ELISpot) assay was used to enumerate the numbers of total and Env-specific IgG secreting plasmablasts and memory B cells in peripheral blood mononuclear cells (PBMCs). Frozen PBMC were thawed and resuspended at 2 x 10^6 cells/ml in 10% FBS RPMI media containing 10% heat-inactivated FBS, 1% Penicillin/Streptomycin and 2% L-Glutamine (Life Technologies, Grand Island, NY, USA) and incubated overnight at 37°C in 5% CO_2 to assess Env-specific plasmablast responses. To assess Env-specific memory B cell responses, PBMCs were resuspended at 1 x 10^6 cells/ml 10% FBS RPMI containing 5ng/ml human recombinant interleukin-2 (rhIL-2, Cat.#3440-10-5X, Mabtech AB, Nacka Strand, Sweden) and 0.5µg/ml imidazoquinoline resiquimod (R848, Cat.#3611-5X, Mabtech AB, Nacka Strand, Sweden) and incubated for 5 day at 37°C in 5% CO_2.
For coating, sterile, white 96-well filter plates with 0.45µm Hydrophobic PVDF membrane (MilliporeSigma, Burlington, MA, USA) were pre-wet with 15µl 35% ethanol for 1 min, washed with sterile water before being coated with 100µl of 10µg/ml of the respective Env protein; AE.A244_D11 gp120 or B.MN_D11 gp120 (provided by Dr. Shelly Krebs, U.S. Military HIV Research Program, Walter Reed Army Institute of Research, Silver Spring, MD, USA). Wells coated with phosphate buffered saline (PBS, Life Technologies, Grand Island, NY, USA) were used as negative control and wells coated with 100µl of 15µg/ml anti-human

IgG antibody MT91/145 (Cat.#3850-3, Mabtech AB, Nacka Strand, Sweden) were used a positive control. Antigen-specific wells and negative control were run in triplicates and positive control in a single well format. Plates were incubated at 4 °C overnight and kept at 4°C for up to 5 days before usage. At the day of the assay, plates were blocked with 10% FBS RPMI media for 30 minutes at room temperature. 2 x 10^5 cells/well and 1 x 10^5 cells/well were added to assess plasmablast and memory B cell responses, respectively and plates were incubated for 16-24 hours at 37°C in 5% CO_2. After incubation, plates were washed with PBS (Thermo Fisher Scientific, Pittsburgh, PA, USA), biotinylated anti-human IgG antibody MT78/145 (Cat.# 3850-6, Mabtech AB, Nacka Strand, Sweden) was added at 1 µg/ml in 0.5% FBS-PBS and plates were incubated at room temperature for two hours. Subsequently plates were washed with PBS, streptavidin-horseradish peroxidase (Mabtech AB, Nacka Strand, Sweden) diluted 1:1000 in 0.5% FBS-PBS was added and incubated for one hour at room temperature. Finally, plates were washed with PBS and 100µl tetramethylbenzidine (TMB) substrate (Mabtech AB, Nacka Strand, Sweden) was added to each well until distinct spots formed when the reaction was stopped using water. Plates were allowed to air dry before counting on CTL ImmunoSpot S5 Core Analyzer using ImmunoCapture software (version 6.3 or higher) and subsequently analyzed using ImmunoSpot software (version 5.0 or higher, Cellular Technologies, Cleveland, OH, USA).
Responses were reported as spot-forming cells (SFC) per million input cells. Positivity was established as at least 20 SFC per million input cells for plasmablast responses and as at least 100 SFC per million input cells for memory B cell response.

Antigen-specific cellular proliferation: The assay was performed as previously described [3]. In brief, frozen PBMCs were thawed as previously described [4] and rested overnight at 37°C, 5% CO2 incubator. The following day PBMC were labeled by incubation with 5 µM CFSE (5-6-carboxyfluoresceindiacetate succimidyl ester; Invitrogen) in 10% HAB media containing RPMI1640 (Gibco), 10% Human AB Serum (HAB; GemCell), 2% L-Glutamine (Gibco) and 1% Penicillin/Streptomycin (Gibco) for 10 minutes at 37°C. Subsequently PBMC were washed and seeded in 10% HAB media at a concentration of 10^6 PBMC/ml and stimulated with 92TH023 Env and Gag LAI peptide pools (JPT Peptides) at a final concentration of 1 µg/ml. PBMC supplemented with DMSO (Sigma), matching the concentration at which antigens were dissolved, were used as a negative control. PBMC stimulated with α-CD3 (30 ng/ml; Clone HIT3a, Cat.# 555336, BD Bioscience) and α-CD28 monoclonal antibody (1 µg/ml; Clone CD28.2, Cat.# 555725, BD Bioscience) served as positive control. If enough PBMC were available stimulation with HCMVpp65 (1 µg/ml; JPT Peptides) served as antigen-specific positive control. After one day of incubation at least 66% viability was used as a cut-off to continue incubation for a total of 6 days. After 6 days PBMC were stained with Aqua Live/Dead (Cat# L34957, Thermo Fisher Scientific) and the following antibodies for 20 minutes at room temperature [5]: anti-CD3 APC H7 (Clone SK7 also known as Leu-4, Cat.# 560176, BD Pharmingen), anti-CD4 BV785 (Clone RPA-T4, Cat.# 300554, Biolegend), anti-CD8 V450 (Clone RPA-T8, Cat.# 560347, BD Horizon), anti-CD27 AF700 (Clone M-T271, Cat.# 560611, BD Bioscience), anti-CD45RO Pe-Cy7 (Clone UCHL-1, Cat.# 337168, BD Bioscience). PBMC were washed and fixed with 1% paraformaldehyde and acquired using a custom-built BD LSR Fortessa (BD Bioscience). Between 100,000 and 150,000 CD3+ lymphocytes were collected for each sample. The average frequency of CFSElow CD4+ T cells of placebo

recipients stimulated with 92TH023 Env peptide pools (background deducted) was used as cut-off.

High-throughput pseudovirus (PSV) neutralization assay
NAb titers were determined using TZM-bl cells (NIH HIV Reagent Program, Division of AIDS, NIAD, NIH, contributed by Dr John C. Kappes, Dr. Xiaoyun Wu and Tranzyme Inc.[6]) in a high-throughput assay utilizing robotic liquid handling. The following PSVs were assessed: a multi-subtype tier 1 PSV panel and murine leukemia virus (MuLV) (nonspecific control). Serum was diluted 1:5 in growth medium and serially diluted using the Biomek NXP liquid handler (Beckman Coulter, Indianapolis, Indiana, USA). Titered serum (12.5 ml/well) was transferred to 384-well culture plates and incubated with an equal volume of PSV for 45 min at 37°C. TZM-bl cells ($3x10^3$ cell/well) with DEAE-dextran (25 mg/ml) were added to each well and incubated for an additional 48 hours. Relative light units were detected with the SpectraMax Paradigm Microplate Reader (Molecular Devices, Sunnyvale, California, USA) using the Bright-Glo Luciferase Assay System (Promega Corporation, Madison, Wisconsin, USA). Neutralization dose–response curves were fitted by nonlinear regression using the LabKey Server, and the final titer is reported as the reciprocal of the dilution of serum necessary to achieve 50% neutralization (50% inhibitory dose).

Fc-mediated effector function assays:
Cell lines and primary cells
The CEM.NKR.CCR5 cell line was obtained from the NIH HIV Reagent Program, Division of AIDS, NIAD, NIH, courtesy of Dr. Alexandra Trkola[7]. THP-1 cells were obtained from MilliporeSigma, Burlington, MA, USA. Both cell lines were cultured in RPMI 1640 (Thermo Fisher Scientific, Waltham, MA, USA), supplemented with 2 mM L-glutamine, 1% penicillin and streptomycin, and 10% heat-inactivated FBS (R-10) at 37°C and 5% CO_2. Peripheral blood mononuclear cells (PBMC), used as effectors, were obtained from apheresis samples collected from HIV seronegative individuals under protocol RV229B/WRAIR #1868 and WRAIR #2567. PBMCs were isolated by density-gradient sedimentation using Ficoll-Paque (Lymphoprep, Nycomed Pharma, Oslo, Norway) and then cryopreserved. Cryopreserved cells from all subjects were stored in liquid nitrogen until used in the assays. White blood cells (WBC) were generated by lysing red blood cells from whole human blood using ACK lysis buffer (ThermoFisher Scientific, Waltham, MA).

Protein coupling to fluorescent beads:
Recombinant gp120 CRF01_AE (CM235, Immune Technology, New York, NY, USA) was biotinylated at a biotin (Cat.# 21338, Thermo Fisher Scientific, Waltham, MA, USA) to gp120 ratio of 50 according to the manufacturer's instructions, and excess biotin was removed using Zeba desalting columns (PI-89883, Thermo Fisher Scientific, Waltham, MA, USA). Biotinylated gp120 was then incubated at a 1:1 ratio with yellow-green (Cat.# F8776, Life Technologies, Carlsbad, CA, USA) or red NeutrAvidin-fluorescent beads (Cat.# F8775, Life Technologies, Carlsbad, CA, USA) for 2 hours at 37°C. Coupled beads were washed and resuspended with 100x volume in 0.1% BSA.

Antibody-Dependent Cellular Phagocytosis (ADCP):

ADCP was measured as previously described[8]. Briefly, 10μl of gp120-coated yellow-green beads were incubated with 100μl of 200-fold diluted plasma samples for 2 hours at 37°C in 96-well polypropylene plates before addition of 25,000 THP-1 effector cells. After 18 hours incubation at 37°C, THP-1 cells were fixed with 4% formaldehyde solution (Cat.# 1008b, Tousimis, Rockville MD USA) and fluorescence was evaluated by flow cytometry. The phagocytic score was calculated by multiplying the percentage of bead-positive cells by the geometric mean fluorescence intensity (gMFI) of bead-positive cells and dividing by 10^4.

Antibody-Dependent Neutrophil Phagocytosis (ADNP):
ADNP was carried out as described in [9]. Briefly, 10μl of gp120-coated red fluorescent beads were incubated with 100μl of 200-fold diluted plasma samples in 96-well polypropylene plates for 2 hours at 37°C, followed by addition of 50,000 white blood cells for 1 hour at 37°C. To identify neutrophils, cells were stained with anti-CD3 AF700 (Clone UCHT1, Cat.# 557943, BD Pharmingen, San Diego, CA, USA), anti-CD14 APC-Cy7 (Clone MφP9, Cat.# 557831, BD, BD Pharmingen, San Diego, CA, USA), and anti-CD66b Pacific Blue (Clone G10F5, Cat.# 305112, Biolegend, San Diego, CA, USA). Cells were then fixed with 4% formaldehyde solution and analyzed by flow cytometry. The phagocytic score was calculated by multiplying the percentage of bead-positive cells by the gMFI of bead-positive cells and dividing by 10^4.

Antibody-Dependent Complement Deposition (ADCD):
ADCD, adapted from [10], was carried out using gp120 CRF01_AE (CM235, Immune Technology) coated CEM.NKR.CCR5 cells. 2μg of gp120 CRF01_AE was added per 10^6 CEM.NKR.CCR5 cells and incubated for 1 hour at room temperature (RT). Protein-coated CEM.NKR.CCR5 cells were washed twice and resuspended in R-10 media. 25,000 cells-gp120 were incubated in 96-well polypropylene plates with heat-inactivated (56°C for 30 min) plasma diluted at 1:10 for 1 hour at 37°C. During this time, lyophilized guinea pig complement (CL4051, Cedarlane, Burlington, Canada) was reconstituted per the manufacturer's instructions in 1 mL cold water, and centrifuged at 13,000 RPM for 5 min at 4°C. The supernatant containing complement was kept on ice for a maximum of 1 hour, until used. Cells were washed with PBS and resuspended in 200 μl of guinea pig complement, which was prepared at a 1:50 dilution in Gelatin Veronal Buffer with Ca^{2+} and Mg^{2+} (IBB-300x, Boston BioProducts, Ashland, MA). After incubation at 37°C for 20 min, cells were washed in 15mM EDTA and stained with a FITC-conjugated anti-mouse complement C3 detection antibody (Clone 6C9, Cat.# CL7631F, Cedarlane, Burlington, Canada). Cells were then fixed with 4% formaldehyde solution and analyzed by flow cytometry. An ADCD score was calculated: ((%FITC+ cells)*(gMFI of FITC+ cells)/10^4.

Antibody-mediated Natural Killer (NK) cell activation:
Recombinant gp120 CRF01_AE (CM235, Immune Technology) was incubated overnight (O/N) at 4°C in flat-bottom MaxSorp plates (Cat.# 44-2404-21, Thermo Fisher Scientific, Waltham, MA, USA) at 150 ng/100 μl/well in PBS. Cryopreserved healthy control PBMC were resuspended at $5x10^5$/mL R-10 with 20 units IL-2/mL (Roche, Mannhein Germany) and incubated O/N at 37°C. On the following day, the gp120-coated plate was washed 4x with 200 μl PBS/well and blocked with 200 μl R-10 for 30 min at RT. 100 μl of 25-fold diluted plasma was added for 2 h at 37°C, followed by 4x wash with 200 μl PBS/well. $2.5x10^5$ PBMC were added with Monensin (eBioscience, San Diego, CA, USA) and Brefeldin A (BD Biosciences,

San Jose, CA, USA) for 5h at 37°C. Cells were washed, transferred to a 96-well U-bottom polypropylene plate, and stained for surface markers: anti-CD3 AF700 (Clone UCHT1, Cat.# 557943, BD Pharmingen, San Diego, CA, USA), anti-CD56 PE-Cy7 (Clone B159, Cat.# 557747, BD Biosciences, San Jose, CA, USA), anti-CD107a PE-Cy5 (Clone H4A3, Cat.# 555802, BD Biosciences, San Jose, CA, USA), anti-CD16 APC-Cy7 (Clone 3G8, Cat.# 561726, BD Biosciences, San Jose, CA, USA), anti-CD19 BV510 (Clone HIB19, Cat.# 302242, Biolegend, San Diego, CA, US), anti-CD14 BV510 (Clone M5E2, Cat.# 301842, Biolegend, San Diego, CA, USA), and live/dead aqua fixable stain (Cat# L34957, Thermo Fisher Scientific, Waltham, MA, USA). Cells were then washed with PBS 2% FBS and fixed for 15 min at RT with Fixation Medium A (Cat.# GAS001S100, Thermo Fisher Scientific, Waltham, MA, USA). Cells were stained intracellularly with anti-IFN-γ V450 (Clone B27, Cat.# 560371, BD Biosciences, San Jose, CA, USA), anti-TNFα FITC (Clone Mab11, Cat.# 502906, Biolegend, San Diego, CA, US), and anti-MIP-1β PE (Clone D21-1351, Cat.# 550078, BD Biosciences, San Jose, CA, USA), prepared in Permeabilization Medium B (Cat.# GAS002S100, Thermo Fisher Scientific, Waltham, MA, USA). After washing with PBS 2% FBS, cells were analyzed by flow cytometry.

Antibody-Dependent Cellular Cytotoxicity (ADCC):
ADCC was measured using the PanToxiLux assay (OncoImmunin, Gaithersburg, MD, USA). PBMC were resuspended at 2×10^6 cells/ml in R-10 and rested overnight at 37°C. Target CEM.NKR.CCR5 cells were incubated with gp120 CRF01_AE (CM235, Immune Technology) at 37°C for 90 min before addition of TFL4 fluorescent target cell marker and NFL1 viability marker. CEM.NKR.CCR5 cells were then washed and resuspended in R-10 at 8×10^5 cells/ml. PBMC effector cells were washed and resuspended at 2.4×10^7 cells/ml in R-10. 25µl of each effector and target cells (effector to target ratio of 30:1) were incubated in 96-well polypropylene plates with 75µl of fluorescent granzyme B substrate for 5 min at RT before addition of 100-fold diluted plasma samples. Cells were then incubated for 1 hour at 37°C, washed and analyzed by flow cytometry.

Trogocytosis:
Trogocytosis was carried out as described in [11]. CEM.NKR.CCR5 cells were washed with PBS and stained with PKH26 (Sigma-Aldrich, St-Louis, MO, USA) at 2µM in Diluant C at RT for 5 min. Cells were then washed with R-10, resuspended in R-10, and incubated with gp120 CRF01_AE (CM235, Immune Technology) for 1 hour at RT in 96-well polypropylene plates. Cells were washed twice with R-10 and incubated with 200-fold diluted plasma samples. Cryopreserved healthy control PBMC were next added in R-10 at an effector to target cell ratio of 50:1 and then incubated for 5 h at 37 °C. After the incubation, cells were washed, stained with live/dead aqua fixable stain and anti-CD11c APC-Cy7 (clone Bu15, Cat.# 337218, Biolegend, San Diego, CA, USA), washed again, and fixed with 4% formaldehyde (Tousimis, Rockville, MD).

Negative and Positive Control Plasma:
Plasma from people living with HIV, collected under protocol RV315B/WRAIR #1749B, was used as a positive control. Commercial normal human serum was obtained from Gemini Bio-Products (West Sacramento, CA, USA). Plasma samples from people living without HIV were collected under protocol WRAIR#1868 and used as a negative control along with commercial

normal human serum. For ADCC, pre-vaccination baseline (Week 0) samples were used to determine the cutoff for positivity. For all other antibody effector function assays, the cutoff for positivity was calculated as: (average of negative control values) + (2 x Standard Deviation).

Flow Cytometry:
Flow cytometry data was collected on the LSRII flow cytometer (Becton Dickenson, Franklin Lakes, NJ, USA) and analyzed using FlowJo Version 10.6.0 software (Becton Dickenson, Franklin Lakes, NJ, USA).

Intracellular cytokine staining:
Cryopreserved PBMCs were thawed, washed, counted, and resuspended in 10%FBS-RPMI at $10x10^6$ cells/ml in 50 mL conical. Any samples that did not meet cell yield and viability criteria, >66%, were excluded from analysis. 100µl of resuspended cells at $10x10^6$ cells/mL and 25µl of each stimulant, co-stimulant, antibodies and transport blockers (DMSO (Cat#D2650-100ML, Millipore Sigma), THO23 ENV, Gag LAI, V2 peptide pools (JPT), SEB, CD28/49d (Clone CD28.2/9F10, Cat #555725/555501, BD), CD154 (Clone TRAP1, Cat# 555701, BD), CD107a (Clone H4A3, Cat# 561348, BD), Brefeldin A (Cat# B7651-5MG, Sigma Aldrich) and monensin (Cat# 554724, BD)) were plated in a 96 well plate and incubated for 6 hours at 37ºC/5% CO_2. Next, plates were centrifuged at 300xg/6mins and washed with 200µL of PBS. The plates were centrifuged, and cells were then resuspended in 50µl of Live/Dead Aqua stain (Cat# L34957, ThermoFisher) or PBS and incubated in the dark, at room temperature for 30 minutes. After incubation, 100µl of Staining Buffer was added to each well and the plate was centrifuged at 300xg/6mins. The cells were washed one more time with Staining Buffer. After the wash, cells were blocked with 100µL of 10% Normal mouse IgG (Cat# 10400C, ThermoFisher)/Staining Buffer for 15min in the dark at room temperature. Then, the plates were centrifuged and the cells were resuspended in 50µl of surface staining cocktail or Staining Buffer and incubated in the dark, at room temperature for 30 minutes. Afterwards, 150µl of Staining Buffer was added to each well and the plates were centrifuged at 300xg/6mins. The cells were washed two more times with 200µL of staining buffer. Cells were then fixed in 200µL of 2% Paraformaldehyde (Cat. # 1008b, Tousimis, Rockville MD USA) in PBS in the dark, at room temperature for 15 minutes. Next, 100µL of Staining Buffer was added to each well and centrifuged at 800xg/6mins. The cells were then resuspened in 200µL of Staining Buffer and the plates were kept at 4C overnight. Once the plates were centrifuged at 800xg/6mins, the cells were resuspended in 200µl of BD 1X Perm/wash Buffer (BD Biosciences) and the plates were incubated for 15 min in the dark at room temperature. Afterwards the plates were centrifuged at 800xg for 6 min and supernatant flicked from the wells. Next, cells were resuspended in 50µl ICS antibody cocktail or BD perm/wash buffer and incubated in the dark, at room temperature for 30 minutes. After the incubation, 150µl of BD 1X perm/wash buffer was added to each well and centrifuged at 800xg/6 mins. Lastly, cells were washed twice with 200µL of Staining Buffer and resuspended in 250µl of Staining Buffer. The cells were run in plate mode on LSRII (BD Biosciences). Commercial antibodies include: AQUA viability dye, CD14 BV510 (clone M5E2, Cat# 301841, Biolegend), CD19 BV510 (clone M5E2, Cat# 302241, Biolegend), CD56 BV510 (clone HCD56, Cat# 318339, Biolegend), CD3 APC-H7 (clone UCHT1, Cat# 641397, BD), CD4 BV605 (clone SK3, Cat# 300556, Biolegend), CD8 PerCP eF710 (cloneRPA-T8, Cat# 46-0087-42, eBioscience), CD107a PE-Cy7 (clone H4A3, Cat# 561348, BD), CD154 PE-Cy5 (clone 24-31, Cat# 555701, BD), Granzyme B AF700 (clone GB11, Cat# 560213, BD), IFN-γ

eF450 (clone B27, Cat# 48-7319-42, eBioscience), IL-2 PE (clone MQ1-17H12, Cat# 559334, BD),), IL-17 PerAF647 (cloneMP4-25D2, Cat# 500818, Biolegend) and TNF-α FITC (clone MAb11, 554512, BD).

COMPASS analysis:

COMPASS uses a Bayesian hierarchical framework to model all observed cell subsets and select those most likely to have antigen-specific responses. Cell-subset responses were quantified by posterior probabilities, and human subject–level responses were quantified by two summary statistics that describe the quality of an individual's polyfunctional response which can be correlated directly with clinical outcome. Raw Boolean data from Flow Jo 9.9.6 was utilized and imputed into R Bioconductor for computation of posterior probabilities and generation of heatmaps.

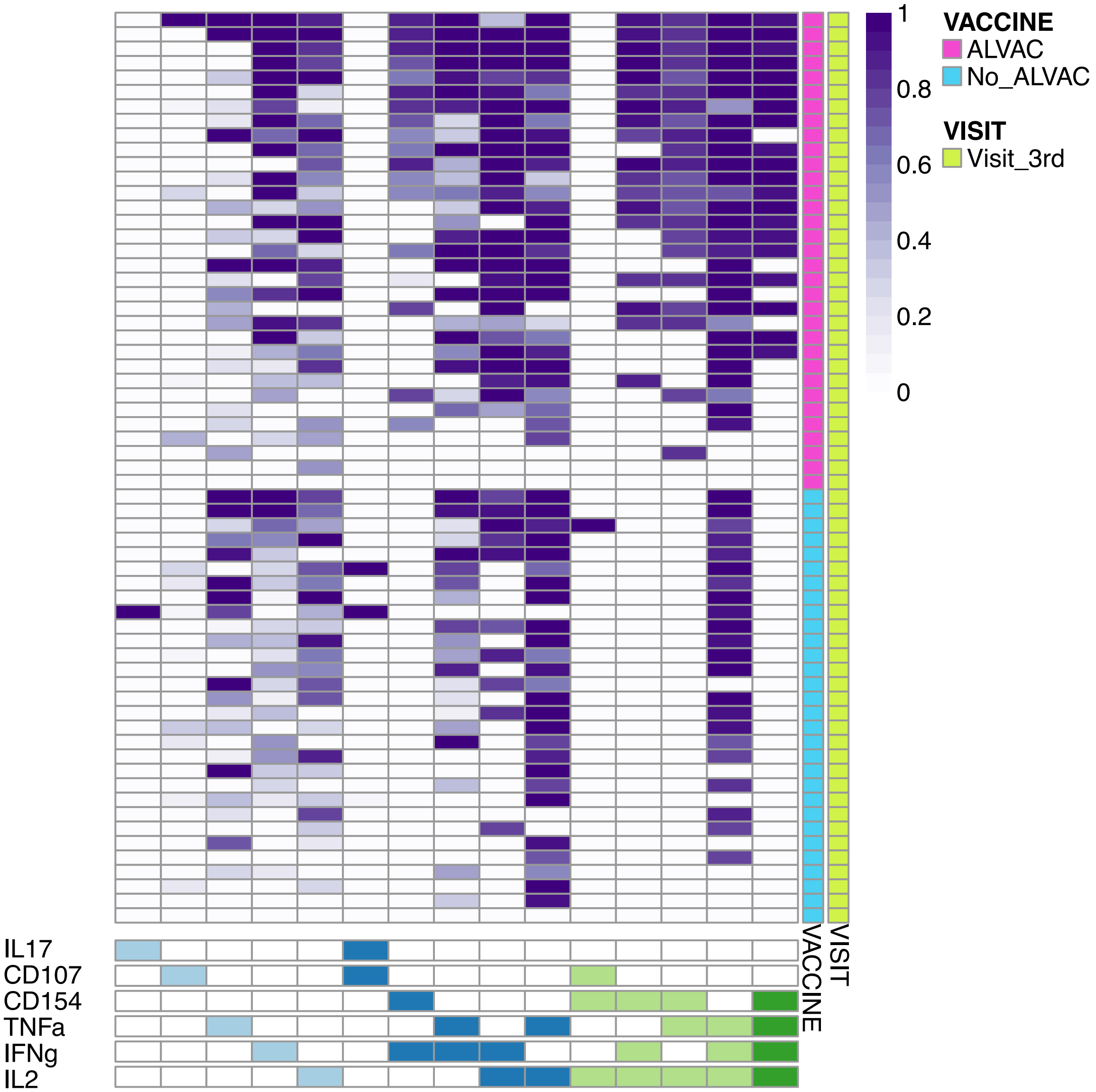

Supplemental Figure 1**.** Heatmap of COMPASS posterior probabilities for Env-specific (TH023) CD4+ T-cells from participants that received ALVAC or not.

Bottom columns correspond to the different cell subsets modeled by COMPASS, color-coded by the cytokines (IL-21, IL-2, TNF-α, IL-4, IFN-γ, CD154) they express (white = "off", shaded = "on", color = "degree of functionality"), and ordered by degree of functionality from one function on the left to six functions on the right. Rows correspond to individual participants. Each cell of the heatmap shows the probability that a given cell-subset (column) has an antigen-specific response in the corresponding participant (column), where the probability is color-coded from white (zero) to purple (one). Env-specific (TH023) CD4+ polyfunctional T-cells were characterized by the expression of CD154 (CD40L), IFN-γ, TNF-α, and IL-2.

References:

1. Karasavvas N, Billings E, Rao M, et al. The Thai Phase III HIV Type 1 Vaccine Trial (RV144) Regimen Induces Antibodies That Target Conserved Regions Within the V2 Loop of gp120. *AIDS Research and Human Retroviruses* 2012; **28**(11): 1444-57.
2. Liu F, Niu Q, Fan X, et al. Priming and Activation of Inflammasome by Canarypox Virus Vector ALVAC via the cGAS/IFI16-STING-Type I IFN Pathway and AIM2 Sensor. *J Immunol* 2017; **199**(9): 3293-305.
3. Pitisuttithum P, Nitayaphan S, Chariyalertsak S, et al. Late boosting of the RV144 regimen with AIDSVAX B/E and ALVAC-HIV in HIV-uninfected Thai volunteers: a double-blind, randomised controlled trial. *Lancet HIV* 2020; **7**(4): e238-e48.
4. de Souza MS, Ratto-Kim S, Chuenarom W, et al. The Thai phase III trial (RV144) vaccine regimen induces T cell responses that preferentially target epitopes within the V2 region of HIV-1 envelope. *J Immunol* 2012; **188**(10): 5166-76.
5. Schuetz A, Deleage C, Sereti I, et al. Initiation of ART during early acute HIV infection preserves mucosal Th17 function and reverses HIV-related immune activation. *PLoS pathogens* 2014; **10**(12): e1004543.
6. Derdeyn CA, Decker JM, Sfakianos JN, et al. Sensitivity of human immunodeficiency virus type 1 to the fusion inhibitor T-20 is modulated by coreceptor specificity defined by the V3 loop of gp120. *J Virol* 2000; **74**(18): 8358-67.
7. Trkola A, Matthews J, Gordon C, Ketas T, Moore JP. A cell line-based neutralization assay for primary human immunodeficiency virus type 1 isolates that use either the CCR5 or the CXCR4 coreceptor. *J Virol* 1999; **73**(11): 8966-74.
8. Ackerman ME, Moldt B, Wyatt RT, et al. A robust, high-throughput assay to determine the phagocytic activity of clinical antibody samples. *J Immunol Methods* 2011; **366**(1-2): 8-19.
9. Karsten CB, Mehta N, Shin SA, et al. A versatile high-throughput assay to characterize antibody-mediated neutrophil phagocytosis. *J Immunol Methods* 2019; **471**: 46-56.
10. Fischinger S, Fallon JK, Michell AR, et al. A high-throughput, bead-based, antigen-specific assay to assess the ability of antibodies to induce complement activation. *J Immunol Methods* 2019; **473**: 112630.
11. Alrubayyi A, Schuetz A, Lal KG, et al. A flow cytometry based assay that simultaneously measures cytotoxicity and monocyte mediated antibody dependent effector activity. *J Immunol Methods* 2018; **462**: 74-82.
